# Microtubule Polymerization Functions in Hypersensitive Response and Accumulation of H_2_O_2_ in Wheat Induced by the Stripe Rust

**DOI:** 10.1155/2016/7830768

**Published:** 2016-08-16

**Authors:** Juan Wang, Yang Wang, Xinjie Liu, Yuanliu Xu, Qing Ma

**Affiliations:** State Key Laboratory of Crop Stress Biology for Arid Areas, Northwest A&F University, Yangling 712100, China

## Abstract

The plant cytoskeleton, including microtubules and microfilaments, is one of the important factors in determining the polarity of cell division and growth, as well as the interaction of plants with invading pathogens. In defense responses of wheat against the stripe rust (*Puccinia striiformis* f. sp.* tritici*) infection, hypersensitive response is the most crucial event to prevent the spread of pathogens. In order to reveal the effect of microtubules on the hypersensitive cell death and H_2_O_2_ accumulation in the interaction of wheat (*Triticum aestivum*) cv. Suwon 11 with an incompatible race, CYR23, wheat leaves were treated with microtubule inhibitor, oryzalin, before inoculation. The results showed that the frequency of infection sites with hypersensitive response occurrence was significantly reduced, and hypersensitive cell death in wheat leaves was suppressed compared to the control. In addition, the frequency and the incidence of infected cells with H_2_O_2_ accumulation were also reduced after the treatment with oryzalin. Those results indicated that microtubules are related to hypersensitive response and H_2_O_2_ accumulation in wheat induced by the stripe rust, and depolymerization of microtubules reduces the resistance of plants to pathogen infection in incompatible interaction, suggesting that microtubules play a potential role in the expression of resistance of wheat against the stripe rust fungus.

## 1. Introduction 

In general, plants are subjected to the attack of a vast number of potential pathogens during their lifetime. As a result, they have evolved intricate defense mechanisms including hypersensitive response (HR) and the accumulation of reactive oxygen species (ROS) [1] to recognize and defend the attack of these invading pathogens. The localized hypersensitive cell death, accompanied by the restriction of pathogen growth, is an ubiquitous expression of plant resistance to pathogens [2]. Typically, HR occurs during successful defense in the host plants, usually leaving only small necrotic spots. Meanwhile, ROS plays important roles in defense response during plant-pathogen interactions [3–5]. Generation of ROS, especially hydrogen peroxide (H_2_O_2_), has been reported as one of the earliest responses of plant cells to the attack of various pathogens [3, 6, 7]. H_2_O_2_ accumulation can inhibit fungal growth [8] and is also involved in the occurrence of HR during the early infection stage [7] as well as regulates a myriad of cellular signaling pathways [9]. Understanding the resistance mechanisms of plants against the invasion of pathogens is critical to develop novel and sustainable disease control approaches.

The plant cytoskeleton, including microtubules and microfilaments, is a highly dynamic subcellular structure that is associated with the plant defense response. For example, cytoskeletal elements are responsible for cytoplasmic aggregation, organelle movements, papilla formation, H_2_O_2_ production, and HR-cell death beneath the infection site [2, 10–12]. Evidence for a crucial role of the cytoskeleton in plant defense has been provided by using drugs that alter the polymerization-depolymerization dynamics of microtubules (colchicine, taxol, or oryzalin) and microfilaments (cytochalasins, latrunculin, or phalloidin). Effects of cytoskeleton inhibitors on defense response of plants during pathogen infection have been studied in several plant-microbe systems. During the interaction between cowpea and cowpea rust fungus,* Uromyces vignae*, cytochalasin treatment greatly delayed the generation of HR [12]. In* Linum usitatissimum*-*Melampsora lini *system, the inhibition of HR was also observed after treatment with antimicrotubule agent oryzalin [13]. Moreover, when wheat cells were attacked by nonhost pathogen* Sphaerotheca fuliginea*, oryzalin treatment inhibited the occurrence of HR and allowed* S. fuliginea* to penetrate and form haustoria in mesophyll cells of the wheat [14]. Interestingly, reorganization of microtubules during defense responses varies in different experimental systems. Microtubules were observed gathering around the infection sites upon fungal infection [12, 13, 15, 16] and even were generally disrupted upon perception of an oomycete infection signal [17, 18]. In contrast, microtubules inhibitors propyzamide and oryzalin did not affect the entry rate of fungi into barley (*Hordeum vulgare*) leaf epidermal cells [19]. So it is difficult to deduce common roles for microtubules during plant-microbe systems.

Wheat stripe rust, caused by* Puccinia striiformis *f. sp.* tritici* (*Pst*), occurs worldwide and is one of the most destructive diseases of wheat in many cool and temperate regions, especially in China [20]. The attack of the rust fungus triggers HR and H_2_O_2_ accumulated in the affected leaf mesophyll cells of the resistant wheat cultivars [21, 22]. It is reasonable to assume that the more we understand the resistance mechanisms of the wheat against the stripe rust, the more likely we are able to find new ways to control the disease. In the present paper, to provide experimental evidence for a role of microtubules, we focus on the effects of oryzalin on hypersensitive cell death and H_2_O_2_ accumulation in the interaction between wheat cultivar Suwon 11 and an incompatible race CYR23 of* Pst*.

## 2. Materials and Methods 

### 2.1. Plant Cultivars and Pathogen

Wheat (*Triticum aestivum *L.) cultivar Suwon 11 and a Chinese race of* Pst*, CYR23, were used in this study. Suwon 11 is highly resistant to race CYR23. The seedlings were grown in 10 cm plastic pots in growth chamber with a 16 h : 8 h (light : dark) photoperiod (60 mmol m^−2^ s^−1^ photon flux density) at 16°C with 60% relative humidity (RH). Seven-day-old seedlings at the primary leaf stage were inoculated with fresh urediniospores of CYR23 using a fine paintbrush. After inoculation, the seedlings were kept at 100% RH in constant dark for 24 h at 12°C before being cultivated in the growth chamber. Specimens of inoculated wheat leaf tissues were taken at 12, 24, 48, 72, and 96 hours after inoculation (hai). Three independent biological replications were collected at each time point.

### 2.2. Treatment with Oryzalin

Oryzalin (Sigma-Aldrich, St. Louis, MO, USA) was used as inhibitor of microtubules [23]. The chemical was dissolved in dimethylsulfoxide (DMSO) as a 100 mmol stock solution, stored at –20°C, and diluted with distilled water prior to use. For inhibitor treatment, 400 *μ*g mL^−1^ oryzalin solution was injected into the primary leaves of seven-day-old wheat seedlings by pressure infiltration with a needleless syringe, and 1% DMSO was used as control treatment. We confirmed that 1% DMSO did not affect fungal development or the penetration efficiency of* Pst* (data not shown). After injection, leaves were inoculated with fresh urediniospores of CYR23. Specimens of inoculated wheat leaf tissues were taken at 12, 24, 48, 72, and 96 hai.

### 2.3. Detection of Inhibitor Effects on Hypersensitive Response

Detection of hypersensitive cell death was carried out using a whole leaf transparent fluorescence staining method [24]. Wheat leaf segments of 3 cm long were clipped from the center of inoculated leaves. Leaf sections were fixed and decolorized in a boiling mixture of lactophenol : ethanol (1 : 2, v/v) for 1.5 min and stored overnight at room temperature (20°C). For Calcofluor staining, the cleared leaf segments were washed twice with 50% ethanol (v/v) for 15 min. The leaves were then rinsed twice with distilled water and soaked in 0.05 M NaOH twice. After washing 3 times with distilled water, the specimens were incubated in Tris-HCl buffer (0.1 M, pH 8.5) for 30 min and then stained with 0.1% (w/v) Calcofluor M2R (Sigma-Aldrich, St. Louis, MO, USA) for 5 min. After washing 4 times (10 min each) with water and once (30 min) with 25% (v/v) aqueous glycerol, cleared leaf segments were mounted on glass slides in microscopy solution and examined with fluorescent microscopy. To investigate the effects of the microtubule depolymerization on the hypersensitive cell death of wheat, the number of penetration sites displaying necrosis was calculated. The formation of substomatal vesicles was defined as a penetration site or infection site. At least 50 penetration sites on each of the four leaf segments were scored for each of the time points. All the specimens were examined under a Nikon 80i fluorescent microscope (Nikon Corporation, Japan).

### 2.4. Detection of Inhibitor Effects on H_2_O_2_

The detection of H_2_O_2_ was analyzed histochemically using the 3,3-diaminobenzidine (DAB; Amresco, Solon, OH, USA) staining method [7, 21]. The inoculated primary leaves were cut and the cut ends were immersed in a solution containing 1 mg mL^−1^ DAB dissolved in HCl-acidified (pH 3.8) distilled water. Leaves were incubated for additional 8 h to allow DAB uptake and react with H_2_O_2_. After incubation, inoculated leaves were cut into 1.5 cm long segments and then fixed and decolorized in boiling 95% ethanol for 10 min before being cleared in saturated chloral hydrate. Subsequently, leaf segments were stored in microscopy solution (50% glycerol) and examined under differential interference contrast (DIC) optics with a Nikon 80i microscope (Nikon Corporation, Japan).

## 3. Results 

### 3.1. Oryzalin Treatment Had No Effect on Infectious Development of* Pst*

Although pharmacological study generally represents a common approach to tackle the role of cytoskeleton in plant-microbe interactions, the anticytoskeletal drugs applied may also damage the microbial cytoskeleton that plays an important role during plant colonization. To determine the effects of oryzalin (400 *μ*g mL^−1^) on the development of* Pst*, we compared the infectious development of* Pst* inoculated on oryzalin treated leaves with that of the control (leaves treated with 1% DMSO).

Both on the control (1% DMSO) and on oryzalin treated leaves, urediniospores germinated normally, and germ tubes grew on the leaf surface until they reached stomas, where the tip of the germ tube swelled and entered into stomatal cavity through stomatal aperture. A substomatal vesicle was formed within the cavity and then developed into 1–3 infectious hyphae. Growth of the infection hyphae made them get in touch with the mesophyll cells, which induced the development of a haustorial mother cell. Our results indicated that treatment with 400 *μ*g mL^−1^ oryzalin solution did not affect the infectious development of* Pst *on wheat leaves.

### 3.2. Oryzalin Treatment Increased the Susceptibility of Resistant Wheat Plants to* Pst*

A few uredia were observed on sites with necrosis in leaves pretreated with the microtubule inhibitor oryzalin (infection type 2 or middle resistance) 15 days after inoculation. However, only some necrotic elongated spots without uredia production were found in control wheat leaves (infection type 0 or nearly immune reaction). This indicated that the resistance level of wheat cultivar Suwon 11 to CYR 23 was decreased upon microtubules depolymerization, suggesting that microtubules may play an important role in the incompatible interaction between wheat and* Pst*.

### 3.3. Oryzalin Inhibited the Hypersensitive Response in Wheat during Wheat-*Pst *Interaction

**Figure 1:**
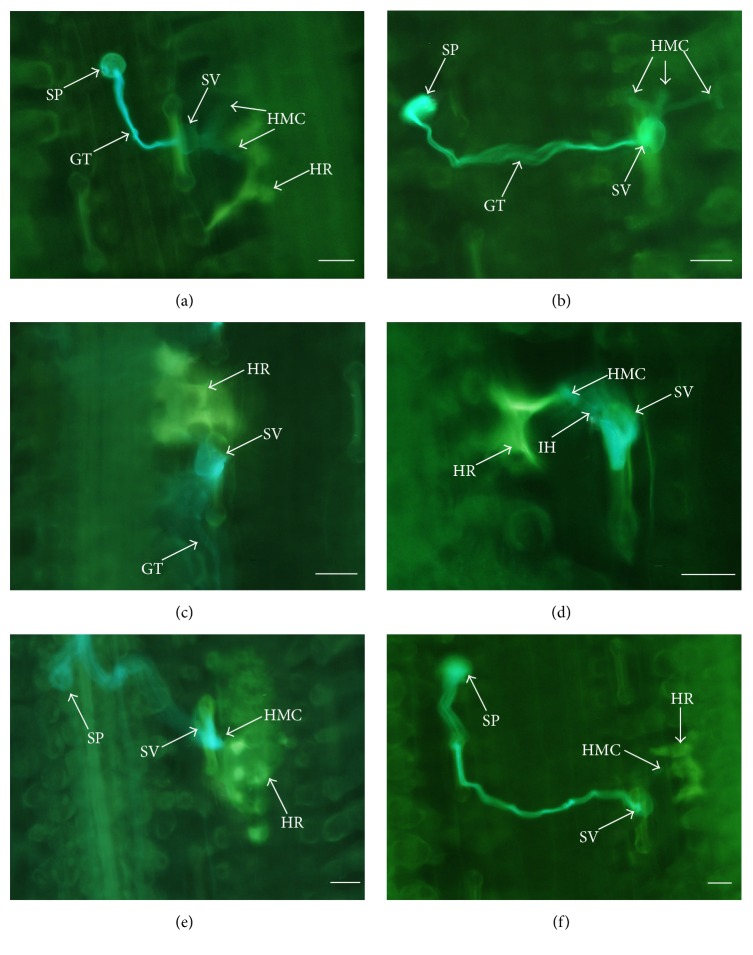
Fluorescence micrographs of hypersensitive cell death localization in incompatible interaction between wheat and* Pst* (race CYR23) in DMSO-only (control) and oryzalin treatments. (a) In control, haustorial mother cells formed and mesophyll cells showed HR reaction, 24 hai. (b) More than two haustorial mother cells formed treated with oryzalin, 24 hai. (c) Conspicuous HR in mesophyll cells was observed in control, 48 hai. (d) The apex of the infection hypha formed a haustorial mother cell. HR was induced by HMC and the whole cells started to lose original shape treated with oryzalin, 48 hai. (e) In control, many HR cells were visualized in mesophyll cells, 96 hai. (f) Slight HR-like cells appeared in the mesophyll cells treated with oryzalin treatment, 96 hai. GT: germ tube; HMC: haustorial mother cell; HR: hypersensitive response; IH: infection hypha; SP: spore; and SV: substomatal vesicle. Scale bars = 50 *μ*m.

In wheat-*Pst* incompatible interaction, the fungal development was remarkedly restricted in infection sites by hypersensitive response of the mesophyll cells. Microscopically, in the control leaves, the HR induced by haustorial mother cells was obvious in mesophyll cells at 24 hai (Figure 1(a)). However, only a few slight fluorescence stainings could be observed at the infection sites in oryzalin treated leaves, and, occasionally, HR could not be detected although three or more haustorial mother cells were formed at infection sites (Figure 1(b)). Although, both in the control and in the oryzalin treated leaves, the ratios of penetration sites with HR were increased significantly at 48 hai in comparison with 24 hai, the extent of HR in mesophyll cells was much less in the treated leaves than that in the control (Figures 1(c) and 1(d)). With incubation time advancing, the number of penetration sites with necrotic mesophyll cells continued to increase, and almost every infection site was necrotic in the control leaves at 96 hai (Figure 1(e)). However, in oryzalin treated leaves with advancing incubation time, 96 hai, less penetration sites with necrotic mesophyll cells were detected, and the extension of necrosis was also smaller than that of the control (Figure 1(f)).

**Figure 2:**
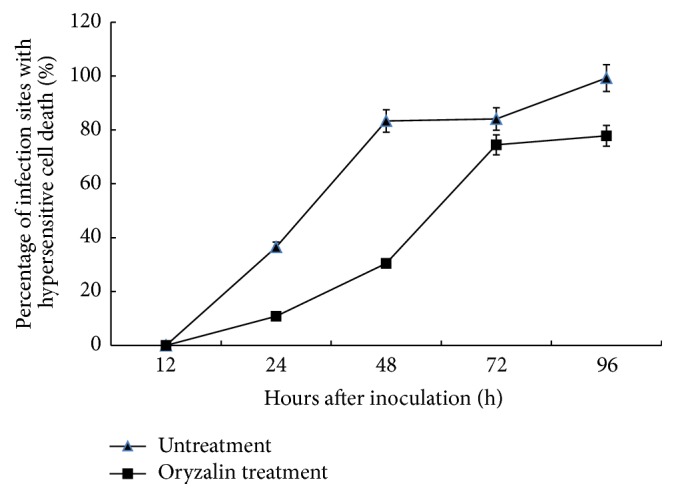
Incidence of mesophyll cells of wheat leaves at infection sites exhibiting hypersensitive cell death after inoculation with* Pst* (race CYR23) in DMSO-only and oryzalin treatments. Bars represent standard deviation. Replicate experiments led to similar results.

The percentage of penetration sites with mesophyll necrotic cells was significantly lower in the oryzalin treatment than in the control over the whole examination period (Figure 2). There were 36% infection sites that had necrosis in the control, but only about 11% in the oryzalin treated specimens at 24 hai. The percentage of incidence of hypersensitive cell death in the control leaves increased rapidly to 83% at 48 hai, followed by a slight increase at 72 hai, and reached approximately 100% at 96 hai. In contrast, in treatment with oryzalin, the percentage of hypersensitive cell death was only 30% at 48 hai but markedly increased to 74% at 72 hai and finally reached 77% at 96 hai. These results showed that hypersensitive response occurrence induced by* Pst* infection was reduced by oryzalin treatment, indicating that normal hypersensitive cell death was suppressed after depolymerization of microtubules in wheat mesophyll cells, especially in the early period of pathogen infection.

### 3.4. Oryzalin Treatment Suppressed H_2_O_2_ Accumulation during Wheat-*Pst* Interaction

**Figure 3:**
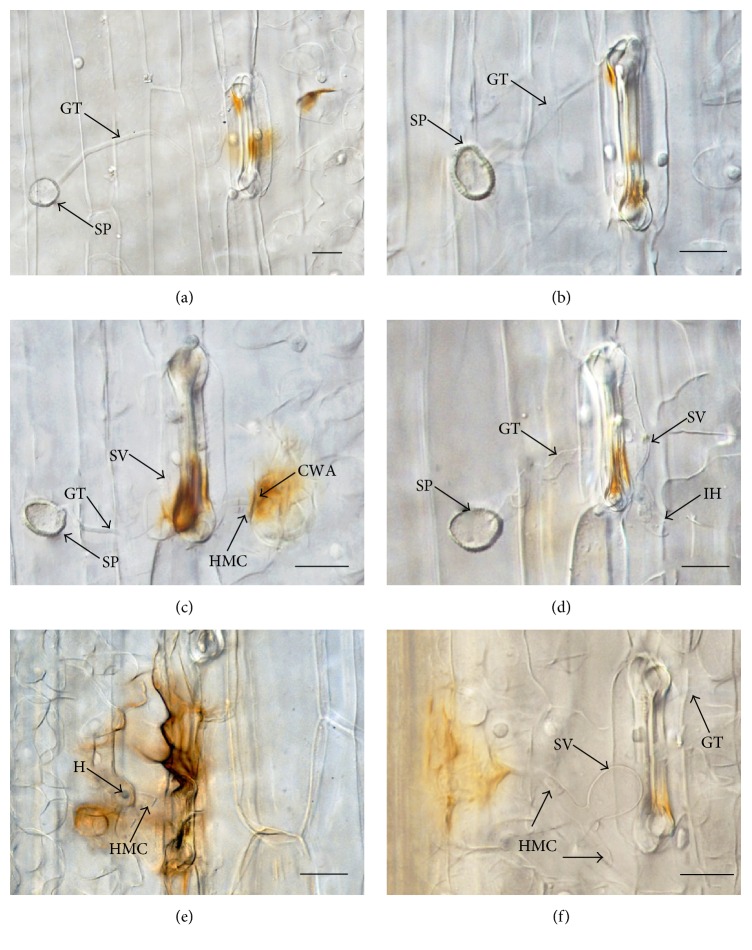
Micrographs of differential interference contrast (DIC) of H_2_O_2_ accumulation in wheat against* Pst* (race CYR 23) in DMSO-only and oryzalin treatments. (a) Mesophyll cells and guard cells showing DAB staining in control, 24 hai. (b) Positive DAB staining was detected mainly in guard cells treated with oryzalin, 24 hai. (c) Guard cells showing obvious and stronger reddish-brown H_2_O_2_ accumulation and mesophyll cells exhibiting the extension of plant cell wall apposition (CWA) in control, 48 hai. (d) Weaker DAB staining detected in guard cells treated with oryzalin, 48 hai. (e) Mesophyll cells and guard cell exhibit intensive H_2_O_2_ accumulation in control, 96 hai. (f) Mesophyll cells and guard cell showed slight H_2_O_2_ accumulation in oryzalin treatment at 96 hai. GT; germ tube; HMC; haustorial mother cell; SP; spore; and SV; substomatal vesicle. Scale bars = 25 *μ*m.

After* Pst* hyphae entering through the opening stomata, in the solvent-only control, H_2_O_2_ accumulation was first observed both in the mesophyll cells and in the guard cells as indicated by reddish-brown staining due to DAB polymerization at 24 hai (Figure 3(a)). Up to 48 hai, stronger reddish-brown DAB staining was detected and more mesophyll cells with DAB staining appeared (Figure 3(c)). At 96 hai, both mesophyll cells and adjacent cells showed strong DAB staining (Figure 3(e)). On the contrary, in oryzalin treated specimens, DAB staining was restricted mainly in the guard cells at 24 hai (Figure 3(b)), and the DAB staining in guard cells became weaker at 48 hai when haustorial mother cells were formed (Figure 3(d)). Although obvious DAB staining was detected both in mesophyll cells and in guard cells at 96 hai, the stain was much weaker than that of the control at the same time point (Figure 3(f)).

**Figure 4:**
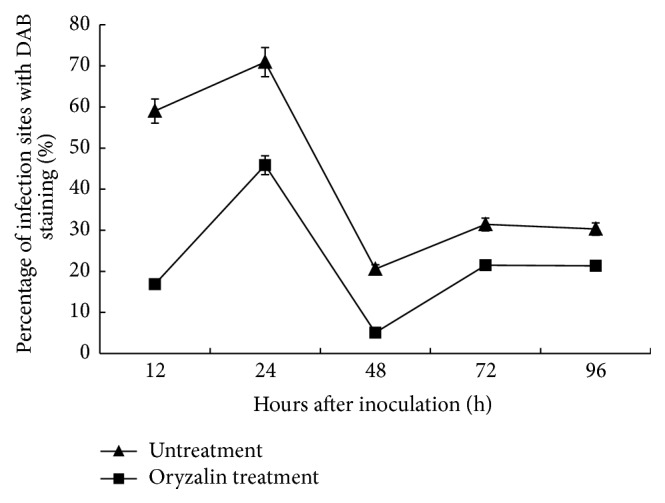
Percentage of mesophyll cells of wheat at interaction sites exhibiting H_2_O_2_ accumulation after inoculation with* Pst* (race CYR23) in DMSO-only and oryzalin treatments. At least 50 infection sites of each of four leaf pieces were scored for each time point. Bars represent standard deviation. Replicate experiments led to similar results.

During the examined time period, the oryzalin treated specimens had significantly lower percentage of penetration sites with DAB staining in the incompatible interaction between Suwon 11 and CYR23 in comparison with the control, although both of them showed similar trends (Figure 4). In the specimens treated with DMSO only, the percentage of infection sites with DAB staining was 60% at 12 hai, reached the peak of approximately 70% at 24 hai, and then decreased sharply to 20% at 48 hai, followed by an increase to 30% at 72 hai, and kept the same level to 96 hai. In contrast to the control, the numbers in the oryzalin treated specimens at the same experimental time points were 17%, 45%, 5%, and 20% (Figure 4). Those results clearly showed that the microtubules depolymerization drug oryzalin suppressed H_2_O_2_ accumulation during wheat-*Pst* interaction.

## 4. Discussion

In this study we found that the microtubule polymerization inhibitor, oryzalin, caused a reduction in the occurrence of hypersensitive response and accumulation of H_2_O_2_ in wheat cultivar Suwon 11 inoculated with the incompatible* Pst *race CYR23, which increased the susceptibility of wheat to the rust fungus compared to normal. In our previous study, we found that cytochalasin A, an inhibitor of actin polymerization, reduced the incidence of hypersensitive cell death and delayed accumulation of H_2_O_2_ in wheat leaves infected with* Pst* [25]. Meanwhile, our results revealed that the cytoskeleton in mesophyll cells has a potential role in HR generation and H_2_O_2_ accumulation and was involved in plant defense responses. Moreover, depolymerizations of microtubules and microfilaments suppressed the defense reactions and promoted the infection of stripe rust fungus in wheat [14, 26], suggesting that intact microtubules and microfilaments networks are necessary for wheat defending invaded the stripe rust fungus.

The microtubule inhibitor oryzalin provides an acceptable approach to study the role of microtubules in plant-pathogen interaction. Our results in this study indicated that depolymerization of microtubules inhibited HR of plant cells in response to pathogen attack. Similarly, the delay of HR after treatment with oryzalin was observed in a range of incompatible plant-pathogen interactions, including cowpea-cowpea rust fungus [12] and flax-flax rust fungus [13]. H_2_O_2_ generation and accumulation during the early infection stage were often associated with early plant defense responses [7]. H_2_O_2_ accumulation was only detected in guard cells before 48 hai in oryzalin treatment specimens instead of 24 hai in the control, which indicated that oryzalin treatment delayed the accumulation of H_2_O_2_ in wheat against rust fungus attack. Meanwhile, we also found that the burst of H_2_O_2_ was restrained after treatment with oryzalin. The data further confirmed that microtubules are necessary for H_2_O_2_ accumulation. In addition, according to the results, oryzalin also inhibited the hypersensitive response in wheat during wheat-*Pst *interaction. Thus, microtubules may play an essential role in resistance response of wheat against the stripe rust.

Moreover, the role of microtubules in HR remains controversial. Oryzalin allowed incompatible oomycete hyphae to spread in the manner of a compatible interaction [27]. However, disruption of microtubules by oryzalin, cell death, and nuclear movements were not affected during the infection of cowpea-cowpea rust fungus [12]. Therefore, we suggest that the role of microtubules in induction of HR varies between different interaction systems.

Traditionally, the plant microtubules are essential players for many different cellular events such as growth, division, cell motility, production of the ER body, vesicular sorting, signal transduction, and cell wall deposition [28]. For the cytoskeleton response to pathogen attack, the role of the microtubules has been reported in different plant-microbe interactions. In barley-*Erysiphe* and flax-*Melampsora* interactions, radial arrays of microtubules formed beneath the appressoria [15, 16]. Treatment with microtubule inhibitors delayed onset of the hypersensitive response in the flax-*Melampsora* system [23]. Moreover, microtubules were identified as a central component in the control of protoplast volume during the response to hyperosmotic stress [29] and the membrane fluidity in cold sensing [30]. In addition, microtubules might act as a negative regulator of ion channel activity or as stress-focusing elements that collect and convey membrane perturbations to a channel [31].

Pathogens are able to suppress the host defenses by secreting effector proteins. In turn, plants evolved resistance proteins, which allow recognition of these effectors. This leads to effector-triggered immunity (ETI) and activation of the hypersensitive response (HR) [32]. ETI or HR involves the production of reactive oxygen species (ROS) and the transcriptional activation of genes, encoding antimicrobial pathogenesis-related (PR) proteins. The signaling pathways of ETI are fine-tuned by plant signaling molecules such as salicylic acid (SA), jasmonic acid (JA), and ethylene (ET) [33, 34]. The hormone SA plays a major role in plant resistance to hemi/biotrophic pathogens [34]. Multiple regulator proteins control microtubule dynamics. Different regulators use different mechanisms to regulate microtubule dynamics. MAP65, a microtubule-associated protein conserved in higher eukaryotes, binds to microtubule to stop microtubule depolymerization [35]. In addition, mutants accumulate in* Arabidopsis thaliana* MAP65-3 increased levels of SA and constitutively express genes encoding PR proteins in the leaves, indicating that AtMAP65-3 exerts a role in negatively regulating plant defense responses [36]. Therefore, the focus of future work in this field should be studying the functions of microtubule-associated proteins in controlling microtubule dynamics that take part in the resistant response of wheat against* Pst*.
